# Imaging and Molecular Mechanisms of Alzheimer’s Disease: A Review

**DOI:** 10.3390/ijms19123702

**Published:** 2018-11-22

**Authors:** Grazia Daniela Femminella, Tony Thayanandan, Valeria Calsolaro, Klara Komici, Giuseppe Rengo, Graziamaria Corbi, Nicola Ferrara

**Affiliations:** 1 Neurology Imaging Unit, Imperial College London, London W12 0NN, UK; g.femminella@imperial.ac.uk (G.D.F.); v.calsolaro@imperial.ac.uk (V.C.); 2 Imperial Memory Unit, Charing Cross Hospital, Imperial College London, London W6 8RF, UK; tony.thayanandan@nhs.net; 3 Department of Medicine and Health Sciences, University of Molise, 86100 Campobasso, Italy; klara.komici@unimol.it (K.K.); graziamaria.corbi@unimol.it (G.C.); 4 Department of Translational Medical Sciences, Federico II University of Naples, 80131 Naples, Italy; giuseppe.rengo@unina.it; 5 Istituti Clinici Scientifici Maugeri SPA—Società Benefit, IRCCS, 82037 Telese Terme, Italy

**Keywords:** Alzheimer’s disease, positron emission tomography (PET), magnetic resonance imaging (MRI)

## Abstract

Alzheimer’s disease is the most common form of dementia and is a significant burden for affected patients, carers, and health systems. Great advances have been made in understanding its pathophysiology, to a point that we are moving from a purely clinical diagnosis to a biological one based on the use of biomarkers. Among those, imaging biomarkers are invaluable in Alzheimer’s, as they provide an in vivo window to the pathological processes occurring in Alzheimer’s brain. While some imaging techniques are still under evaluation in the research setting, some have reached widespread clinical use. In this review, we provide an overview of the most commonly used imaging biomarkers in Alzheimer’s disease, from molecular PET imaging to structural MRI, emphasising the concept that multimodal imaging would likely prove to be the optimal tool in the future of Alzheimer’s research and clinical practice.

## 1. Introduction

Alzheimer’s disease (AD) is a neurodegenerative disease that is responsible for 60–80% of all cases of dementia worldwide. Recent epidemiological data indicate that approximately 5.7 million Americans of all ages are living with AD in 2018 and 10.5 million people were suffering with dementia in Europe in 2015. The prevalence of dementia in Europe ranges from 4.7% to 6.8% [1]. Estimated projections suggest that by 2025, the number of people over 65 with AD will reach 7.1 million in the U.S., which is almost a 29 percent increase from the 2018 prevalence, and by 2050 the population affected will grow to 13.8 million, posing a great burden on health systems [2]. Clinically, AD is typically characterised by impairment in short-term memory to such an extent as to interfere with activities of daily living, while later symptoms include impairment in the other cognitive domains, such as language, orientation, judgment, executive functions, behavioural changes, and, ultimately, motor difficulties.

The first criteria proposed for AD diagnosis were developed in 1984 and focused only on clinical symptoms. However, the exceptional amount of research conducted since has helped clarify that the phase of dementia in AD is preceded by a long preclinical phase of several decades that evolves through a continuum, with the prodromal stage of mild cognitive impairment (MCI), and ultimately leads to dementia [3]. In this long preclinical phase, an early diagnosis can be made with the help of biomarkers. Based on this evidence, the National Institute on Aging (NIA) and the Alzheimer’s Association in 2011 published new guidelines incorporating biomarker tests in addition to clinical symptoms, moving from a symptom-based definition to a biology-based definition of AD [4].

The biology of AD is characterised by two major protein abnormalities in the brain of affected individuals: the extracellular accumulation of amyloid β (Aβ) plaques and intraneuronal deposits of neurofibrillary tangles (NFTs). Insoluble Aβ plaques are formed of aggregated Aβ peptides that derive from the abnormal cleavage of the amyloid precursor protein (APP) into hydrophobic Aβ peptides. Aβ is thought to be the trigger or the driver of the disease process, mainly based on evidence from familial AD cases, leading to the amyloid hypothesis of AD [5]. NFTs are composed of hyperphosphorylated tau protein aggregates which accumulate in the neuron cytoplasm, leading to destabilisation of microtubules and axonal transport [6]. Both proteinopathies can trigger oxidative stress, microvascular dysfunction, and blood–brain barrier (BBB) disruption, and can induce the activation of an inflammatory response within the brain, ultimately resulting in neuronal damage and consequent neurodegeneration [3].

All these pathological changes that manifest at earlier or later phases of the AD continuum can now be explored with the use of biomarkers, some of which are still only used in a research framework and are awaiting clinical validation. Overall, the main biomarkers in AD can be broadly divided into cerebrospinal (CSF) and imaging biomarkers. Research is ongoing in the field of blood biomarkers, but large clinical studies are needed to assess their diagnostic potential [7]. In this review, we will focus on imaging biomarkers, both those currently available in clinical practice and those that are only part of the research framework [8]. Over CSF biomarkers that constitute an indirect measure of the ongoing pathological processes, imaging ones have the advantage of providing information on the in vivo pathological processes, giving a “window” to the changes happening in the brain at the different stages of the disease, and are less invasive and troublesome for the patients. We will focus on neurodegenerative imaging biomarkers (MRI and glucose metabolism), amyloid and tau imaging, and the newest in vivo biomarkers for neuroinflammation and BBB dysfunction.

## 2. Imaging of Neurodegeneration

### 2.1. Structural Magnetic Resonance Imaging (MRI)

Atrophy seems to be an unavoidable, inevitable progressive component of neurodegeneration. Brain tissue loss correlates well with cognitive deficits, both cross-sectionally and longitudinally in AD [9]. Structural brain changes are accurately consistent with upstream Braak stages of neurofibrillary tangle deposition [10,11] and downstream neuropsychological deficits [12]. Rates of change in several structural measures, including whole-brain [13], entorhinal cortex [14], hippocampus [15], and temporal lobe volumes [16], correlate closely with changes in cognitive performance, validating atrophy in these regions as markers of AD. For atrophy markers to be useful clinically, the subtleties should be known at the different stages of the disease, and their relationship with other imaging and biological markers should be understood. Atrophy measures change with disease progression over AD disease severity. Structural markers are more sensitive to change than are markers of Aβ deposition, both in MCI and in the moderate dementia stage of AD [17]. However, studies have shown that in the earliest forms of MCI, amyloid burden shows more abnormalities that are structural changes [18,19]. Atrophy is accompanied by microstructural changes, such as axonal loss and metabolite changes, all of which are measured with techniques other than MRI.

Structural MRI is still one of the most widely used neuroimaging techniques in the diagnosis of AD. T1-weighted scans are the most commonly used due to their ability to provide good contrast between grey and white matter and to detect subtle changes in grey matter. MRI gives the best spatial resolution of any clinical neuroimaging technique, so measures from an MRI include grey matter volume, cortical thickness, and volumetric measures of the hippocampus [20]. Measurements of grey matter are usually done visually, but recently there has been increased use of automated methods to calculate volume and cortical thickness [21,22] and subcortical segmentation of the hippocampus [23,24]. However, with these recent advances in more methodological techniques, visual reading is still the method most often used clinically to read an MRI. This shows the lack of a standardised protocol or method for the diagnosis of AD but is of high interest for researchers [24,25]. Clinicians also use structural MRI to determine whether cognitive impairment is due to reasons other than AD such as tumours or subdural hematomas [26].

**Figure 1:**
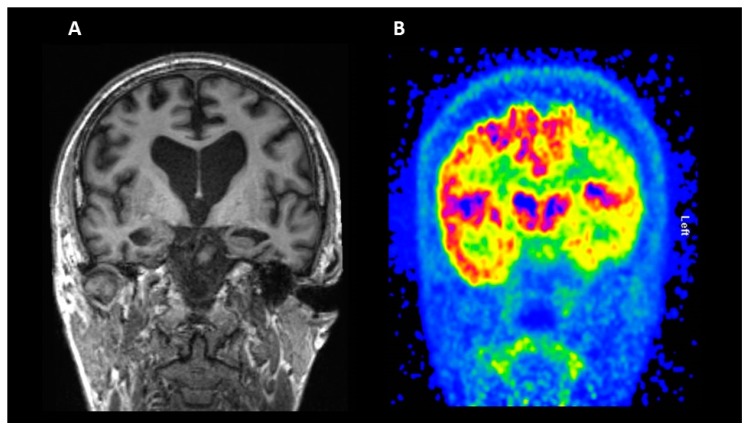
Imaging biomarkers of neurodegeneration. Coronal structural MRI section (panel **A**) and 18F-fluorodeoxyglucose (FDG) PET (panel **B**) from a patient with Alzheimer’s disease (AD).

Structural MRI studies have shown reduced hippocampal volume in individuals with amnestic MCI, and its reduction is thought to be one of the most predictive and sensitive measures of AD [27]; however, studies have shown other neuropsychiatric disorders such as schizophrenia [28] and depression [29] demonstrate a reduction in hippocampal volume as well. Figure 1, panel A, shows a coronal structural MRI session where hippocampal atrophy is shown (left larger than right). Therefore, the implementation of MRI-based biomarkers for clinical use requires validation across both clinical and analytical techniques. The diagnostic and prognostic accuracy of neuroimaging markers are dependent on both how the biomarker is measured (visual or quantitative) and which one is measured (MRI, Amyloid PET, fluorodeoxyglucose (FDG)-PET, etc.) [30]. Variation in methods and scanners can introduce noise and bias into the data which can impact the diagnostic accuracy. 

### 2.2. Fluorodeoxyglucose (FDG) PET

Glucose is the main source of energy used by the brain, which consumes around 25% of the amount circulating the whole body. The cerebral glucose metabolism is regulated by transport through the BBB, led by glucose transporters (GLUTs); GLUT1 is the main transporter on the BBB, while GLUT3 is the main transporter on neuron membranes, with a higher efficacy than GLUT1 [31]. GLUT1 is also present on astrocytes, which can uptake glucose in response to neuronal secretion of glutamate and produce lactate, another source of energy for neuronal activity [32]. The glucose consumption rate in the brain can be displayed in vivo using the PET tracer 18F-FDG, which reaches the neurons and enters the glycolytic process until the formation of FDG-6-phosphate, which will then stay trapped in the cells, at the same rate as the glucose [31]. The glucose consumption is not only an indicator of synaptic activity, whose loss is one of the main features of AD [33], but also reflects the excitatory glutamate release and recycling between astrocytes and neurons [34]. A reduction in the glucose metabolism is recognised as a biomarker of neurodegeneration, appearing years before the cognitive symptoms [33,35]. A pattern of reduced [18F]FDG uptake in posterior cingulate, hippocampi, and medial temporal structures is typical in AD and MCI, with subsequent spreading to the whole cortex as the disease progresses [33] (see Figure 1, panel B), while cerebellum, visual and primary motor cortices, and basal ganglia nuclei are less affected [36]. A different pattern of hypometabolism can be seen in other variants of AD, like posterior cortical atrophy and primary progressive aphasia [37]. It is interesting to note that the glucose hypometabolism is correlated with cognitive impairment and its severity, while the results of studies evaluating the same correlation between amyloid load and severity of cognitive impairment are less homogeneous [38,39]. The reduction in glucose metabolism in regions like the precuneus and posterior cingulate has been demonstrated to be associated with the severity of cognitive impairment [38]. A large study evaluated the baseline cerebral metabolic rate for glucose (CMRgl) in 298 subjects from the ADNI cohort (142 aMCI, 74 pAD, and 82 controls), correlating it with cognitive impairment severity; both the disease groups showed a reduction in the CMRgl in posterior cingulate, precuneus, and frontal and parietotemporal cortices compared with the cognitively intact subjects [40]. The CMRgl rate in the left frontal and temporal cortices was significantly correlated with low Mini-Mental State Examination (MMSE) scores when evaluating only the AD population [40]. In a different study, the pattern of regional hypometabolism appeared to be associated with specific cognitive domains, with visuospatial ability impairment correlated to a reduced metabolism in the posterior regions and impairment in language abilities with a left hemisphere reduction [38]. Interestingly, the impact of cognitive reserve in AD has also been studied with FDG-PET: Ewers et al. evaluated an ADNI cohort of cognitively normal subjects, classified as preclinical AD or healthy control based on the biomarkers profile, and they found that a higher level of education was associated with reduced FDG-PET in the amyloid-positive group [41]. This finding is in line with the literature, supporting the theory that high cognitive reserve can compensate the biological impairment, and highly educated subjects can show a degenerative profile worse than expected for the symptoms [41].

The accuracy of FDG-PET compared to serial clinical evaluation relative to post mortem pathological diagnosis was evaluated in a cohort of 44 subjects grouped as AD and not AD [42]. This study demonstrated that, in the diagnostic process, the FDG-PET is superior to clinical evaluation, which reached the same diagnostic power only later on in the follow-up [42].

Several studies demonstrated that FDG-PET is also a good predictor of disease progression from MCI to AD, according to a few longitudinal studies [43,44]. A longitudinal study aiming to establish the sensitivity and specificity of FDG-PET in patients evaluated and followed up for dementia proved an FDG-PET sensitivity of 93% in detecting progressive dementia and a specificity of 76%; it was also able to distinguish patients with AD from patients with other degenerative diseases with a sensitivity of 94% and a specificity of 73% for AD and 78% for other diseases [45]. It is also worth noticing that a negative scan at baseline indicates an unlikely progression across 3 years [45]. The use of FDG-PET in the clinical setting for the diagnostic process of MCI is more debated, with some studies showing hypometabolism in the cortex and others being inconclusive in the identification of MCIs [31]. In 2015, a Cochrane meta-analysis of 14 studies, for a total of 421 subjects, aimed to evaluate the effectiveness of FDG-PET in identifying MCI subjects converting to dementia at the follow-up [46]. According to the authors, the result of the meta-analysis did not support the use of FDG-PET in routine clinical use in MCI subjects. A limitation of this meta-analysis was the poor methodological quality of some of the studies, leading to risk of bias; therefore, more uniform protocols would be required to get to a satisfactory conclusion [46]. However, the use of FDG-PET is of high value in the diagnostic process, especially in the most difficult cases [37]. Few retrospective studies have actually demonstrated the usefulness of the FDG-PET in clarifying the diagnosis and increasing the cholinesterase inhibitor prescription; moreover, in atypical or uncertain cases, a repeated follow-up FDG-PET improved the diagnostic power and management [37]. FDG-PET is a widely used imaging technique, both in research and clinical settings, with a high predictive value and diagnostic power for Alzheimer’s disease and different types of dementia. Together with the other biomarkers, such as cortical atrophy and amyloid and tau deposition, it is a fundamental tool for early diagnosis, selection criteria, and follow-up evaluation in clinical trials.

## 3. Amyloid Imaging

Accumulation of Aβ fibrils in the form of amyloid plaques is a neuropathological hallmark for autopsy-based diagnosis confirmation of dementia caused by AD [47]. Aβ deposition is thought to precede cognitive symptoms in AD and is therefore a potential preclinical marker of disease [48]. There have been different approaches to noninvasively visualise amyloid deposition in human brains with amyloid PET radiotracers. Typically, amyloid imaging agents bind to insoluble fibrillary forms of Aβ 40 and Aβ 42 deposits, which are major components of compact neuritic plaques and vascular deposits.

Clinical criteria for the suitable use of amyloid imaging in patients demonstrate the need to integrate scanning with detailed clinical and cognitive evaluations. These criteria state that amyloid imaging should only be used under certain circumstances such as in patients with persistent or progressive unexplained cognitive impairment or unclear clinical presentations [49]. Amyloid imaging, as stated by the clinical criteria, should not be used to determine severity of dementia or in patients with probable AD and of typical age, with a family history of dementia, and/or with the presence of the APOE4 allele [50,51]. 11C-Pittsburgh Compound B (PiB) was the first amyloid imaging PET agent used in human subjects in 2002 [52]. However, the PiB compound is labeled with 11C, with a short half-life of only 20 min, limiting its use. To overcome this problem, 18F-labeled Aβ tracers, with a longer half-life of 110 min, are used to show reliable assessment of brain amyloid in a single 15-minute scan. There are only three approved Aβ tracers for clinical use: 18F-Florbetapir [53], 18F-Florbetaben, and 18F-Flutemetamol.

18F-Florbetapir was the first tracer approved for the detection of in vivo amyloid and the first 18F-labelled tracer approved by the FDA since Fludeoxyglucose (FDG); subsequently, this has become the most widely used amyloid tracer. Multicentre studies showed that a high Aβ burden on 18F-Florbetapir PET was associated with poor memory performance in healthy participants [54]. It has also been shown that approximately 50% of MCI patients had a high Aβ burden on 18F-Florbetapir PET [55]. In phase III studies, 18F-Florbetapir demonstrated high sensitivity and specificity (92% and 100%, respectively) in detecting Aβ pathology with no tracer retention in control subjects [56,57]. 18F-Florbetaben reveals a high affinity for fibrillary Aβ in brain homogenates, selectively labelled Aβ plaques, and cerebral amyloid angiopathy in tissue sections from patients with AD [58]. 18F-Florbetaben PET can also detect Aβ pathology in a wide spectrum of neurodegenerative conditions such as frontotemporal lobar degeneration (FTLD). Cortical retention of 18F-Florbetaben was higher in patients with AD than in healthy controls or patients with frontotemporal dementia [59]. 18F-Flutemetamol, in phase I and II studies, was able to differentiate between patients with AD and healthy controls [60,61]. The prediction of progression to AD in patients with MCI was improved when combined with measures of brain atrophy [62]. The tracers discussed above have high affinity and selectivity for fibrillar Aβ in plaques and other Aβ-containing lesions [63,64]. When Aβ PET scans are visually read, cortical tracer retention is usually higher in patients with AD than in healthy controls, particularly in the frontal, cingulate, parietal, and lateral temporal cortices. Both visual and quantitative assessments of amyloid scans from different stages of disease progression reveal a consistent pattern of tracer retention that coincides with amyloid deposition found post mortem in patients with sporadic AD [65]. Longitudinal studies have shown that minute increases in Aβ deposition can be measured using PET; however, these changes can only be seen in those who have either have high or low burdens [66]. Acceptable Aβ loads in normal individuals have also been observed, and approximately 7% of these individuals have an increase of Aβ within 2.5 years above the threshold for “normal” levels [67]. 

The pivotal use of Aβ imaging is facilitating differential diagnosis in patients who present with atypical symptoms of dementia [68]. Clinical presentations of FTLD can be difficult to differentiate from early onset AD. FTLD does not have Aβ pathology, and these patients, for the most part, show no cortical retention of 11C-PiB—another amyloid tracer [69,70,71]. Therefore, using amyloid PET can help differentiate between FTLD and AD. The patterns of Aβ deposition can also help differential diagnosis. Patients with cognitively stable Parkinson’s disease (PD) have no cortical Aβ deposition; however, Parkinson’s disease dementia (PDD) shows signs of Aβ deposition [72,73]. 

## 4. Tau Imaging

Tau imaging is the latest innovation in the early detection of neurodegenerative proteinopathies. In the past few years, a number of first-generation tau-selective PET tracers have been developed. 18F-flortaucipir, 18F-THK5351, 18F-THK5317, and 11C-PBB3 have all been extensively used in research studies but have yet to be used clinically. Through imaging studies, tau tracer retention shows an affinity to not only known distributions of aggregated tau but also to mirror patterns of neuronal injury detected by FDG-PET [74,75]. FDG uptake and 18F-THK5317 retention show a negative correlation, primarily in frontal areas [76]. FDG also shows a mediating role in the association between tau pathology and cognitive decline in AD [77]. 

Tau imaging could be very useful to predict progression of AD due to the relationship between tau deposition, cognitive impairment, and neuronal injury. Tau imaging has the ability to assess the regional distribution and density of tau deposits in the brain which could also help with disease staging. While Aβ imaging studies indicate that total Aβ deposition in the brain is more important than regional differences in predicting cognitive decline, tau imaging data suggest that the topographical distribution of tau in the brain is more important than the total level of tau in the brain [78,79]. A combination of tau and Aβ imaging could be highly beneficial in predicting cognitive decline and neurodegeneration. Studies have demonstrated that high levels of cortical tau deposition in those with Aβ pathology showed increased cognitive impairment in several domains [80,81]. 

Most, if not all, applications of tau and amyloid imaging are used for the same purpose: accurate and early detection of AD pathology, disease staging, predicting disease progression, and use in disease-specific clinical treatment trials. However, several groups have suggested that tau imaging is better for disease staging and predicting progression than amyloid imaging [82,83]. These groups have compared patients with AD and non-AD tauopathies and have found significant differences in tracer retention between healthy controls, patients with AD, and patients presenting with atypical AD [84,85]. Interestingly, clinical presentations of patients with atypical AD significantly matched their tau deposits as assessed by 18F-flortaucipoir but not their Aβ burdens as assessed by 11C-PiB [86].

However, studies show that high levels of tau found in specific regions of interest (mesial and temporal lobes) are not found alongside a high level of Aβ. Conversely, high levels of tau are highly associated with high Aβ levels in the neocortex. This suggests that detectable levels of cortical Aβ deposits precede levels of cortical tau deposition. Post mortem studies have shown tau deposits in the mesial temporal cortex in elderly individuals, both healthy and with dementia [87]. These findings suggest that hippocampal tauopathy is age related, and not dependent on but magnified by Aβ pathology [74]; this is now known as primary age-related tauopathy (PART) [88]. 

The in vivo relationship between 18F-flortaucipir and grey matter intensity shows a negative correlation as measured by MRI in healthy controls. Moreover, a study by Wang et al. [89] showed that amyloid plaques affected the association between 18F-flortaucipir retention and cerebral atrophy. Amyloid-positive patients showed a significant association between tau imaging and volume loss, which suggests tau deposition and neuronal loss. 

The best use of tau imaging would be a combination of amyloid imaging and selective tau imaging to explain whether Aβ accelerates or causes the spread of tau outside the mesial temporal cortex. This could also help elucidate whether this spreading into cortical areas corresponds clinically to the development of MCI [74,90].

Much like amyloid imaging, tau imaging can be used for differential diagnosis for neurodegenerative Aβ-related conditions such as Dementia Lewy Body (DLB) and other tauopathies such as progressive supranuclear palsy [91]. Also, approximately 40% of FTLD cases are caused by hyperphosphorylated tau, labelled FTLD-tau. As stated previously, Aβ deposition is not a pathological feature of FTLD; therefore, the tau imaging can help with correct diagnosis, especially for specific forms of the disease [92].

A low hippocampal signal has been observed in some tau tracers which is compounded by the unreliable and irregular tracer binding to the choroid plexus, which just lies above the hippocampus. Researchers have suggested that the tracers bind to the aggregated tau in the choroid plexus [93] despite the lack of in vitro autoradiographic studies showing a consistent failure of tracer binding [94]. Another theory suggests that the tracers actually bind to other β-sheet aggregated proteins, such as iron or transthyretin [95,96]. At the moment, no tau tracers have been validated for clinical use [97], and some researchers highlight the inconsistencies between the in vitro and in vivo binding profiles of the tracers [98].

Something that is even more alarming is the doubt over tau selectivity from some PET tracers. Studies show a there is “off-target” binding resulting from tracer binding to alternative targets. Selegiline, a selective and irreversible inhibitor of monoamine oxidase B, also known as MAO-B, can cause signal reductions in cortical and basal ganglia in 18F-THK5351 imaging. In fact, a single 5 mg dose of selegiline can cause signal reductions of up to 50%. This suggests that a certain percentage of tau binding seen in 18F-THK5351 is caused by MAO-B binding [99]. Newer second-generation tracers, such as 18F-RO69558948, have shown less off-target binding [100] with two other tracers (18F-MK6240 and 18F-PI2620) showing no off-target binding [101,102].

## 5. Imaging of Neuroinflammation

Neuroinflammation refers to the innate inflammatory response of the central nervous system (CNS) to any neuronal insult, such as infections, vascular lesions, trauma, and the presence of abnormal protein aggregates [103]. Data from studies conducted in the last decades indicate that in neurodegenerative diseases, and particularly in AD, neuroinflammation is not only an epiphenomenon secondary to Aβ and tau abnormalities, but it is an essential part of the disease pathophysiology. Results from genome-wide association studies indicate that many of the newly identified genetic risk variants associated with AD involve genes that play an important role in immune function [104]. The cellular players of inflammatory response in the brain are primarily microglia and astrocytes. Microglia activation and reactive astrocytosis can be evaluated in vivo by the use of PET imaging. Thus, in vivo detection of neuroinflammation could represent a useful tool to further clarify the role of immune response in AD pathology and to assess the effectiveness of novel treatments targeting neuroinflammation [105]. 

### 5.1. Imaging Microglia

Microglia are mononuclear resident phagocytes ubiquitously distributed in the brain, where they account for 10%–15% of non-neuronal cells [106]. Microglia are of myeloid lineage, originating from progenitors formed in the yolk sac, and their differentiation occurs in the CNS [107]. Under physiological conditions, microglial cells scan the brain parenchyma continuously in order to maintain the homeostasis and, in doing so, present in a ramified morphology. In this resting state they also provide supportive factors to tissue integrity and secrete trophic factors that help maintain neuronal plasticity [108]. Upon detection of any pathological triggers, mediated by membrane receptors, microglia become activated and migrate to the area of the lesion. They change their shape to an amoeboid one and start releasing proinflammatory cytokines, such as tumour necrosis factor-α and interleukin-1β, and free oxygen radicals, such as nitric oxide and superoxide [109]. Both post mortem and preclinical data indicate that in AD the accumulation of Aβ plaques is the main trigger for neuroinflammation. Activated microglia surround Aβ plaques in an attempt to phagocyte them or degrade them through the secretion of proteolytic enzymes [110,111]. Although the initial microglial activation aims at clearance of Aβ plaques and might exert a neuroprotective effect, its continuous triggering and the inefficacy in the clearing process might lead to a vicious cycle of sustained chronic inflammation, with an ultimately neurotoxic effect [112]. This dual function of microglia has been exemplified in the M1/M2 theory, which postulates that microglia switch from a M1 proinflammatory phenotype to a M2 anti-inflammatory one [113]. However, this theory seems to be over-simplistic, and it is likely that microglial phenotype switching and its dual function are a dynamic process. 

Once activated, microglia express the Translocator Protein 18 kDa (TSPO), formerly known as peripheral benzodiazepine receptor (PBR). In physiologic conditions, TSPO expression is low within the CNS, primarily confined to endothelial cells, ependyma, choroid plexus, olfactory bulb, and glial cells. Following any brain injuries, TSPO expression on microglial outer mitochondrial membrane markedly increases, making it a suitable marker of glial activation [114]. Over the last decades, several TSPO radioligands have been developed, the most widely used being [11C]-PK11195. This tracer was initially used as a racemate, but the R-enantiomer has a greater affinity for TSPO than the S-enantiomer, and subsequent studies only used [11C]-(R)-PK11195 to investigate neuroinflammation in vivo [109,115]. Although [11C]-(R)-PK11195 has been widely used in several neurological diseases associated with neuroinflammation [116,117], this tracer suffers major limitations, such as a poor signal-to-noise ratio due to high nonspecific binding, high plasma protein binding, and the use of [11]C, which limits its use to PET research centers and hospitals with an on-site cyclotron. These difficulties led to the development of second-generation TSPO ligands, with higher TSPO affinity and better kinetics, such as [11C]-PBR28, [11C]-DAA1106, [18F]-DPA714, [18F]-FEPPA, and [18F]-GE180. However, the binding affinity of second generation TSPO tracers is affected by a single-nucleotide polymorphism (SNP) rs6971 in the TSPO gene, which causes an Alanine-to-Threonine substitution in the protein. Based on this, individuals are classified into high-affinity binders (HABs), mixed-affinity binders (MABs), and low-affinity binders (LABs), so that genotyping is essential for appropriate tracer quantification [118].

In AD, Cagnin et al. were the first to report an increase in [11C]-PK11195 binding in the temporal lobe [119], while other groups found no differences between AD patients and controls [120]. Using second-generation TSPO radioligands, other researchers have demonstrated a significant increase in AD subjects with [11C]-DAA1106 [121], [11C]-PBR28 [122], and [18F]-FEPPA [123]. The relationship between microglial activation and amyloid deposition in AD has also been evaluated, finding clusters of significant correlation in most cases [124,125]. Combined PET studies provided evidence for a significant inverse correlation between microglia activation and glucose metabolism in AD patients [126] as well as with hippocampal volume [127]. When looking at cognitive function, the results are varied: some authors have found a significant inverse correlation between TSPO binding and Mini-Mental State Examination (MMSE) scores [124,128], others found no correlation [125], and another group found a positive correlation between the global cortical index and MMSE score [122]. Using different cognitive measures, a negative correlation has been observed between [11C]-PBR28 binding in the inferior parietal lobule and performance on Block Design [123], as well as between [18F]-FEPPA binding in the parietal and prefrontal cortices and visuospatial tasks [129]. A PET multitracer study has recently demonstrated significant widespread correlation between levels of microglial activation and tau aggregation in both MCI and AD subjects, suggesting that these pathologies increase together as the disease progresses. Moreover, microglial activation and amyloid load were also correlated, with a different spatial distribution. The three processes seem to be often found in similar areas of the association cortex [130]. Results are more controversial in the MCI population: some studies have reported increased [11C]-PK11195 uptake in 38% of MCI subjects, while others have shown no differences compared to healthy controls. Similarly, using second-generation radioligands, Yasuno et al. showed significant increases in [11C]-DAA1106 binding in the cerebellum, medial prefrontal cortex, parietal cortex, lateral temporal cortex, anterior cingulate cortex, and striatum in MCI [129], while Kreisl et al. found no differences between MCI patients and controls using [11C]-PBR28 [122].

There are only few studies that have evaluated the longitudinal changes in microglial activation in the AD continuum. Fan et al. demonstrated that microglial activation detected by [11C]-PK11195 increases in AD as the disease progresses, while it is reduced in MCI [131]. A recent study on prodromal AD or MCI subjects using [11C]-PBR28 reported increased longitudinal binding in patients but not in controls, on average equal to 2.5%–7.5% per year [132]. In a study of 64 AD patients, significantly higher global cortical [18F]-DPA-714 binding has been demonstrated in slower decliners compared to fast decliners, further substantiating the concept that early microglial activation could be protective [125].

### 5.2. Imaging Astrocytes

Astrocytes are star-shaped glial cells, conventionally divided in two categories: protoplasmic astrocytes, located in the grey matter, and fibrous astrocytes, located in the white matter. Their main function is to provide nutritional support to neurons and insulate synaptic connections, regulating extracellular concentrations of ions and neurotransmitters. When activated, astrocytes increase the expression of the glial fibrillary acidic protein (GFAP), and the process of reactive astrogliosis aims at neuroprotection. In AD, it seems that astrocytes play an important role in the clearance of Aβ, and after exposure to Aβ they can release cytokines, interleukins, and reactive oxygen species, contributing to the neuroinflammatory process [106].

During neuroinflammation, monoamine oxidase B (MAO B) is up-regulated in reactive astrocytes, and can be targeted in vivo using different PET tracers, such as [11C]-deuterium-l-deprenyl-[11C]-DED- and [11C]-deprenyl-D2 [105]. In a study on AD subjects and amyloid-positive MCI, increased [11C]-DED binding was observed in the frontal, parietal, and temporal cortices, and regional correlation between [11C]-DED uptake and amyloid burden was reported [133]. Results from a multitracer PET study using [11C]-DED, [11C]-PIB, and [18F]-FDG in genetic and sporadic AD patients showed divergent patterns of amyloid deposition and astrocytosis, with the latter process being elevated in the early presymptomatic stages of the disease, and the former increasing with disease progression [134]. Astrocytosis has also been imaged using ligands for the I2-imidazoline receptor, such as [11C]-BU99008. Studies with this PET tracer are underway in AD and MCI subjects.

**Figure 2:**
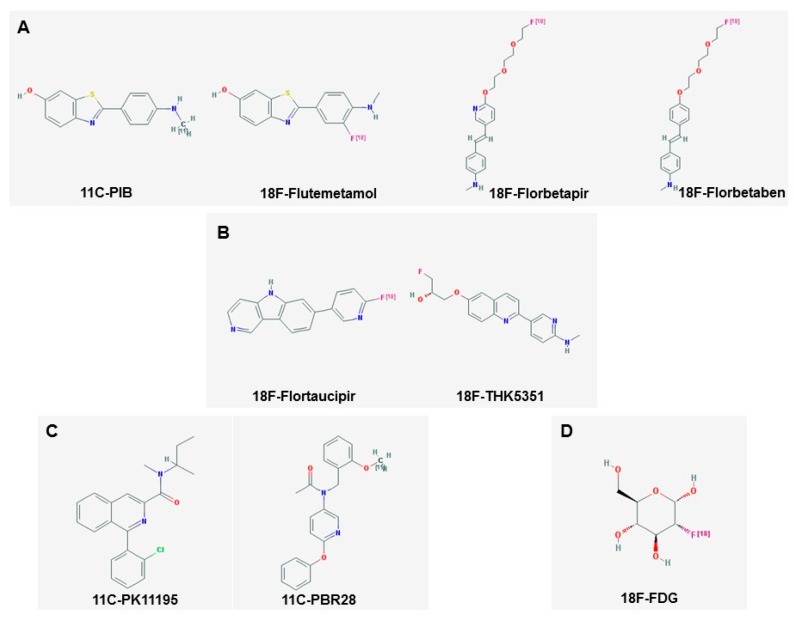
Chemical structure of some PET tracers. Panel **A** shows amyloid PET tracers, Panel **B** shows Tau tracers. Microglial tracers are shown in Panel **C**, and 18F-FDG is shown in Panel **D** (structures downloaded from [135]).

Figure 2 shows the chemical structures of some of the most commonly used PET tracers mentioned so far [135].

## 6. Imaging of Blood–Brain Barrier Dysfunction

The blood–brain barrier (BBB) is a highly functional, specialised barrier separating the intravascular system from the neurons, representing a fundamental interface between circulating cells in the bloodstream and the neuronal system. The BBB operates as part of the neurovascular unit (NVU), a multilayer barrier formed by endothelial cells expressing tight junction proteins, a basal lamina of extracellular proteins, astrocyte end-feet, and pericytes [136]. While low permeability is the usual state of the BBB, where protein and cell transport is led by the tight junction proteins (TJPs) and transporters [136], a breach in integrity and impaired function is a common finding in several diseases [137]. In AD, the deposition of Aβ fibrils in the vessel elicits the release of pro-inflammatory cytokines, contributing to BBB damage and an increase in its permeability [137]; moreover, cerebral amyloid angiopathy affects smooth muscle cells, pericytes, and endothelial cells, increasing the damage [137]. The timing of the BBB disruption and AD progression has been widely studied: an indirect measure of BBB breach is the CSF albumin index, demonstrating structural disruption in Alzheimer’s and vascular dementia [138]. Post mortem studies reported BBB damage in subjects with AD, demonstrating the accumulation of several proteins in the hippocampus and the cortex and the degeneration of pericytes [139]. AD is also characterised by vascular changes in the endothelial and smooth muscle cells, partially secondary to amyloid toxicity; around amyloid deposits in the vessels, endothelial cells are less viable, and microvascular cerebral tissues showed reduced mitochondrial content and a higher concentration of pinocytotic vesicles [140]. 

While in case of tumour, strokes, or inflammatory diseases like multiple sclerosis the breach in the BBB permeability is to a major extent, in dementia it is more subtle and requires specific MRI imaging sequences [136] since other imaging techniques (PET and CT) failed to demonstrate any difference between patients with dementia and healthy controls [141,142]. A study conducted with a PET tracer [68Ga]ethylene-diamine-tetraacetic acid ([68Ga]EDTA) did not demonstrate a difference in CNS permeability between a small group of AD subjects and healthy controls [142]. Similarly, a CT study with meglumine iothalamate failed to show any difference in BBB abnormality between AD and HC [141]. The measurement of BBB permeability with MRI is based on the use of paramagnetic contrast agent Gadolinium-based compounds and the measurement of its leakage from the intravascular space. The techniques used are either dynamic susceptibility contrast-enhanced MRI (DSC-MRI) or dynamic contrast-enhanced MRI (DCE-MRI) [136]. Very few studies have been conducted on small cohorts of AD or MCI patients. Degeneration of the BBB has been demonstrated in the hippocampus with the ageing process; however, that has been seen to appear earlier in subjects with Mild Cognitive Impairment when compared with cognitively intact subjects [139]. This evaluation was conducted using a DCE-MRI; with this technique, grey and white matter regions were simultaneously analysed. In a different MCI population compared to HC, DCE-MRI showed a lower contrast enhancement and slower contrast decay, respectively indicating lower vascular volume and higher BBB permeability in the hippocampi, suggesting impairment in the vasculature and possible BBB disruption [140]. Interestingly, the same difference was not seen in the cerebellum, but, considering that the cerebellum is spared by AD pathology, this is not surprising [140]. 

To investigate if the leakage could contribute to AD, a pilot study with a dynamic contrast-enhanced MRI was conducted on a population of MCI due to AD and early AD by Maastricht ad Leiden Universities [143]. The imaging protocol was designed with a resolution able to separate the vessel filling from the leakage. The authors also evaluated the relationship between BBB permeability and cognitive performance. The results of the study demonstrated a significantly higher BBB leakage rate in the patient group compared to the controls in the grey matter; the leakage volume was significantly higher in the grey matter, in the normal-appearing white matter, and in the cortex [143]. Considering all the subjects together, the leakage volume in the deep gray matter was higher when the MMSE was lower; a significantly higher leakage volume in the deep gray matter was found in the MCI group when compared to the controls. The overall results of the study supported the theory of BBB impairment as a contributing factor to the AD pathology, especially considering the association with the cognitive performance and the early phases of the subjects enrolled [143]. A case control MRI study was conducted on a cohort of 15 AD subjects and 15 healthy volunteers; for this dynamic contrast-enhanced MRI, regions of interest in the deep grey matter, cortical grey matter, white matter, CSF, and carotid and basilary arteries were selected [144]. In this study, the BBB permeability across the two groups did not differ significantly; however, a difference was seen in the temporal pattern after the injection, suggesting an early occurrence of the BBB permeability difference between healthy control and AD subjects [144]. Others have demonstrated that BBB permeability is increased in major dementia disorders but does not relate to amyloid pathology [145]. 

The evaluation of BBB damage and permeability is an interesting challenge, especially considering the complexity of the analysis required; certainly, more studies are needed to develop a reliable MRI protocol acquisition, and robust data results are necessary to be able to apply the technique on the larger scale of the clinical setting.

## 7. Limitations and Future Perspectives

Early diagnosis of sporadic neurodegenerative conditions can be very difficult, especially when patients present with nonspecific symptoms that can be attributable to any form of dementia or neurodegenerative disease. Recently, the NIA-AA research framework criteria for AD have developed the concept that diagnosis should be made based on the measurement of integrated biomarkers, moving towards a more biological definition of the disease. These biomarkers not only concern the presence of Aβ but also must include the tau status of the individual [146]. As the diagnostic criteria for AD continue to develop, the use of amyloid and tau PET imaging is likely to be at the forefront of use in clinical practice. 

It is important to note, however, that PET imaging bears several methodological limitations, from the poor resolution of the PET itself to the presence of brain atrophy, which is certainly a crucial feature to be considered in AD imaging. Particularly regarding the latter, unfortunately, there is a lack of homogeneity in the approach to the atrophy [147]. Some studies’ approach considered the partial volume effect, proportionate to the atrophy, applying partial volume correction; this has been done with different toolboxes or codes [148,149] and for different tracers [148,149,150,151,152]. Some other studies included the grey matter volume as a covariate in the analysis or excluded relative atrophy. The poor spatial resolution of the PET is also a limitation, especially when analysing small areas or areas where the cortical thickness and the voxel area have similar dimensions [147]. A possible solution is the use of combined PET and MR, which allows a better anatomical accuracy and a partial volume correction to the PET findings [153]. Another factor limiting broader and more routine use of PET imaging is the methodological quantification of the signal. Different techniques have been used in research, with various advantages and caveats to be considered. The standardised uptake value (SUV) technique, applicable to static images, is simple and practical to use; however, it is subjective to different variables, such as the tracer uptake time, dose measurement, and receiving body characteristics. More accurate estimates certainly come from kinetic parameters analysis; this is, however, less practical to use, requiring dynamic images and arterial input [154]. Despite the advances in imaging techniques, no single biomarker is likely to be able to provide the diagnostic certainty needed for early detection of neurodegenerative diseases. Identification or diagnosis requires a multimodal approach that combines biochemical and neuroimaging markers of pathology and neurodegeneration [155]. These biomarkers have now been incorporated into the new diagnostic criteria for the prodromal, preclinical, and overt stages of AD [156,157]. Furthermore, AD-specific interventional trials have been able to implement short-duration trials with smaller samples sizes due to the use of Aβ and/or tau biomarkers to confirm target and treatment efficacy [158]. Interpretation of amyloid positivity through PET is done either visually or quantitatively. Amyloid positivity is defined based on the presence of absence of tracer uptake in brain cortical regions compared to the cerebellum due to a lack of amyloid accumulation in this region. Visual analysis is usually performed using a binary scale while quantitative analysis involves receiver operating characteristic analysis without prespecified cut-off values. This causes data to over-fit which could result in sensitivity and specificity values that are overly optimistic [159]. Conversely, visual interpretation is dependent on the reader’s experience, and while most scans are read by multiple readers to confirm positivity or negativity, this is against everyday clinical practice and will have an effect on diagnosis. 

Multimodality imaging is the way forward in both research and clinical contexts in AD, suggesting that a combined use of MRI and PET may increase the accuracy of diagnosis due to the ability to detect pathological brain changes associated with AD in the earliest of stages (Table 1 and Table 2). Moreover, in a research setting, and in particular in clinical trials with drugs targeting biomarkers, multimodal imaging also has the added value of allowing the monitoring of potential side effects of experimental drugs, which could be hindered by the cognitive impairment [160]. Even though in the diagnostic process of a neurodegenerative disease the results of imaging techniques have to be related to the clinical picture, there are some images with a very strong diagnostic power on their own, such as hippocampal atrophy for AD or DaTScan for Lewy Body Dementia [161]. However, studies have shown that high amyloid load or grey matter atrophy is not enough to give a clear predictive sign of AD, with many healthy individuals showing no signs of AD even with the hallmarks of the neuropathological changes [162,163]. The lack of actual multitracer studies, conducted longitudinally and exploring all the biomarkers at the same time-point, needs to be addressed, together with a strictly homogenous methodological protocol, to better facilitate a more detailed insight into disease pathology. Interest is now increasing in the use of plasma biomarkers for global organ diseases, which may be relevant in neurodegenerative disease, especially considering the link between nutrition, diet, and ageing. In particular, genomic, lipidomic, and proteomic biomarkers are increasingly interesting [164]. In particular, the study of genomics, i.e., the calorie-sensitive gene Sirt1, related to lipidomic and proteomic biomarkers, could be a sensitive tool in the assessment of a few chronic diseases which have showed association with AD (such as obesity and diabetes) [165]. Also, plasma biomarkers, due to their easy access, hold potential in terms of early diagnosis. Plasma Aβ levels seem to correlate with cognitive function and with CSF biomarkers [166], and the combination of clinical, imaging, and plasma markers can predict progression in MCI subjects [167]. This once again highlights the need for a clearer diagnostic route that does not rely solely on neuroimaging biomarkers.

## Figures and Tables

**Table 1:** PET tracers in AD.

Target	Tracer	Clinical Correlates in AD	Ref.
Amyloid-β	18F-Florebetapir	Has demonstrated high sensitivity and specificity (92% and 100%, respectively) in detecting Aβ pathology	[13,14]
	18F-Florbetaben	High affinity for fibrillary Aβ, selectively labelled Aβ plaques, and cerebral amyloid angiopathy in tissue sections from patients with AD	[15]
	18F-Flutemetamol	In phase I and II studies, was able to differentiate between patients with AD and healthy controls	[17,18]
Tau protein	18F-flortaucipir, 18F-THK5351, 18F-THK5317, 11C-PBB3	Bind to neurofibrillary tangles with high selectivity and high signal-to-background ratio. Used for early detection of nerve fiber lesions in patients with AD	[39]
Microglial activation	11C-PK11195	Used to investigate neuroinflammation in vivo. There is an increase of binding in the temporal lobe of AD patients.	[73,74]
	11C-DAA1106, 11C-PBR28, 18F-FEPPA	Inverse correlation between microglia activation and glucose metabolism in AD patients as well as with hippocampal volume	[80,81]
	18F-DPA-714	Showed significantly higher global cortical binding in slower AD decliners compared to fast decliners	[79]
Astrocytes	[11C]-deuterium-l-deprenyl-[11C]-DED, [11C]-deprenyl-D2	In AD and amyloid-positive MCI, increased binding was observed in the frontal, parietal, and temporal cortices and regional correlation between 11C-DED uptake and amyloid burden	[59,86]
Glucose Metabolism	18F-FDG	Reduced uptake in posterior cingulate, hippocampi, and medial temporal structures is typical in AD and MCI, with a subsequent spreading to the whole cortex as the disease progresses. The reduction in glucose metabolism in regions like precuneus and posterior cingulate has been demonstrated to be associated with the severity of the cognitive impairment	[121,126]

**Table 2:** MRI correlates in AD.

Target	Sequences	Clinical Correlates in AD	Ref.
Blood–brain barrier (BBB)	Dynamic susceptibility contrast-enhanced MRI (DSC-MRI)	Degeneration of the BBB has been demonstrated in the hippocampus with the ageing process; however, that has been seen to appear earlier in subjects with MCI when compared with cognitively intact subjects	[91]
	Dynamic contrast-enhanced MRI (DCE-MRI)	Significantly higher BBB leakage rate in AD compared to controls in the grey matter; the leakage volume was significantly higher in the grey matter, in the normal-appearing white matter, and in the cortex	[95]
Brain atrophy	Three-dimensional (3D) T1-weighted magnetisation-prepared rapid acquisition gradient-echo (T1-MPRAGE) sequence	Structural brain changes are accurately consistent with Braak stages of neurofibrillary tangle deposition and neuropsychological deficits. Rates of change in several structural measures, including whole-brain, entorhinal cortex, hippocampus, and temporal lobe volumes, correlate closely with changes in cognitive performance, validating atrophy in these regions as markers of AD.	[98,99,100,101,102,103,104]
